# Gene expression and metabolite profiling of *Populus euphratica *growing in the Negev desert

**DOI:** 10.1186/gb-2005-6-12-r101

**Published:** 2005-12-02

**Authors:** Mikael Brosché, Basia Vinocur, Edward R Alatalo, Airi Lamminmäki, Thomas Teichmann, Eric A Ottow, Dimitar Djilianov, Dany Afif, Marie-Béatrice Bogeat-Triboulot, Arie Altman, Andrea Polle, Erwin Dreyer, Stephen Rudd, Lars Paulin, Petri Auvinen, Jaakko Kangasjärvi

**Affiliations:** 1 Plant Biology, Department of Biological and Environmental Sciences, University of Helsinki, P.O. Box 65, Viikinkaari 1, FIN-00014 Helsinki, Finland; 2 The Robert H Smith Institute of Plant Sciences and Genetics in Agriculture, Faculty of Agricultural, Food and Environmental Quality Sciences, The Hebrew University of Jerusalem, Herzl Street, Rehovot 76100, Israel; 3 Institute of Biotechnology, University of Helsinki, P.O. Box 56, Viikinkaari 4, FIN-00014 Helsinki, Finland; 4 Institut für Forstbotanik, Georg-August-Universität Göttingen, Büsgenweg 2, 37077 Göttingen, Germany; 5 AgroBioInstitute, 8 Dragan Tzankov Boulevard, 1164 Sofia, Bulgaria; 6 UMR INRA-UHP Ecologie et Ecophysiologie Forestières, Faculté des Sciences, F-54506 Vandoeuvre, France; 7 UMR INRA-UHP Ecologie et Ecophysiologie Forestières, IFR 110 Génomique, Ecophysiologie et Ecologie Fonctionnelle, INRA Nancy, Route d'Amance, F-54280 Champenoux, France; 8 Turku Centre for Biotechnology, BioCity, Tykistökatu 6, FIN-20521 Turku, Finland

## Abstract

**Background:**

Plants growing in their natural habitat represent a valuable resource for elucidating mechanisms of acclimation to environmental constraints. *Populus euphratica *is a salt-tolerant tree species growing in saline semi-arid areas. To identify genes involved in abiotic stress responses under natural conditions we constructed several normalized and subtracted cDNA libraries from control, stress-exposed and desert-grown *P. euphratica *trees. In addition, we identified several metabolites in desert-grown *P. euphratica *trees.

**Results:**

About 14,000 expressed sequence tag (EST) sequences were obtained with a good representation of genes putatively involved in resistance and tolerance to salt and other abiotic stresses. A *P. euphratica *DNA microarray with a uni-gene set of ESTs representing approximately 6,340 different genes was constructed. The microarray was used to study gene expression in adult *P. euphratica *trees growing in the desert canyon of Ein Avdat in Israel. In parallel, 22 selected metabolites were profiled in the same trees.

**Conclusion:**

Of the obtained ESTs, 98% were found in the sequenced *P. trichocarpa *genome and 74% in other *Populus *EST collections. This implies that the *P. euphratica *genome does not contain different genes *per se*, but that regulation of gene expression might be different and that *P. euphratica *expresses a different set of genes that contribute to adaptation to saline growth conditions. Also, all of the five measured amino acids show increased levels in trees growing in the more saline soil.

## Abstract 2

A *Populus euphratica *DNA microarray was constructed and used to analyze gene expression in trees growing in the desert. *P. euphratica *is shown to express a set of genes that is different from other *Populus *trees and these genes contribute to adaptation to saline growth conditions.

## Background

Most studies on biotic and abiotic stress in plants are performed under controlled laboratory and/or greenhouse environments. The advantage of this approach is that the influence of a single or a few factors affecting the plant can be studied separately in great detail. The disadvantage is that a plant growing in its natural habitat is unlikely to experience only a single stress factor. Furthermore, short term laboratory experiments may not allow plants to acclimate to the imposed constraints as happens during moderate and long lasting constraints in natural habitats. Thus, knowledge obtained from controlled experiments may not be directly applicable to field conditions. The importance of adaptation and acclimation is even more crucial in trees that have a life span of several decades or longer and thus will be exposed to repeated episodes of abiotic and biotic stresses.

Over the past decade, several species in the genus *Populus *have emerged as model woody plants. The genus *Populus *includes a wide variety of species (about 30) from different areas around the world displaying a range of different growth characteristics and tolerance towards various stress conditions [1]. Extensive genetic resources, including expressed sequence tag (EST) collections in several *Populus *species, their relatively small genome (approximately 520 Mbp) and rapid early growth, and the complete sequencing of the whole genome of *Populus trichocarpa *[2] make it possible to perform molecular research in several *Populus *species with an array of tools that are still not available for any other tree species.

*Populus euphratica *Oliv. is considered to be salt tolerant when compared to other *Populus *species [3]. Salinity is a major abiotic stress acting primarily as an osmotic stress and causes the disruption of homeostasis and ion distribution in the cell. In addition, it causes oxidative stress, damage to membranes and proteins, and activates signaling cascades leading to changes in gene expression [4,5]. *P. euphratica *has a natural distribution extending over semi-arid areas in the Middle East and Asia. It grows at locations with a wide variety of temperature and soil conditions, such as high temperatures in the air and high salt content in the soil. *In vitro *experiments have indicated that *P. euphratica *can tolerate up to 450 mM NaCl [6].

Deserts represent one of the harshest ecosystems on earth, combining low precipitation and extreme temperatures, and sometimes also elevated salt levels. Molecular mechanisms of adaptation to desert conditions have previously been studied in the desert legume *Retama raetam *[7,8]. *R. raetam *uses a strategy where the upper, light exposed parts of the plants enter dormancy and protect the lower part of the plant by giving shade [7]. Although *P. euphratica *trees do grow in saline arid desert areas, it does not show physiologically significant drought stress, suggesting access to water [9], and is actually highly sensitive to hydraulic dysfunctions in the xylem [10]. Thus, the strategies used by *P. euphratica *to grow under desert conditions are likely to be different from those used by *R. raetam*, probably relying on access to deep water tables.

Here we describe sequencing of ESTs from *P. euphratica *growing in the Ein Avdat canyon in the Negev desert in Israel [11]. This collection is expected to contain important ESTs for physiological acclimation and adaptation to 'desert' conditions, for example, high temperature, salinity and drought. Although several EST collections are available from multiple tissues in *Populus *[12-19], almost all these collections were produced from plants growing under close to optimal environmental conditions. Thus, ESTs representing genes responsive to abiotic stress, in particular salt and drought, are likely to be under-represented in them. To obtain a more complete collection of the *Populus *abiotic stress-related transcriptome, we sequenced ESTs from normalized *P. euphratica *cDNA libraries, and subtracted cDNA libraries prepared from multiple abiotic stress treatments, including salt, drought, ozone, cold, freezing and flooding. A *P. euphratica *DNA microarray containing 8,153 ESTs representing 6,340 different genes was constructed and used to analyze gene expression in leaf samples from the desert valley Ein Avdat in Israel. In addition, we also performed metabolite profiling on Ein Avdat leaf samples.

## Results

### Normalized and subtracted cDNA library construction

**Figure 1:**
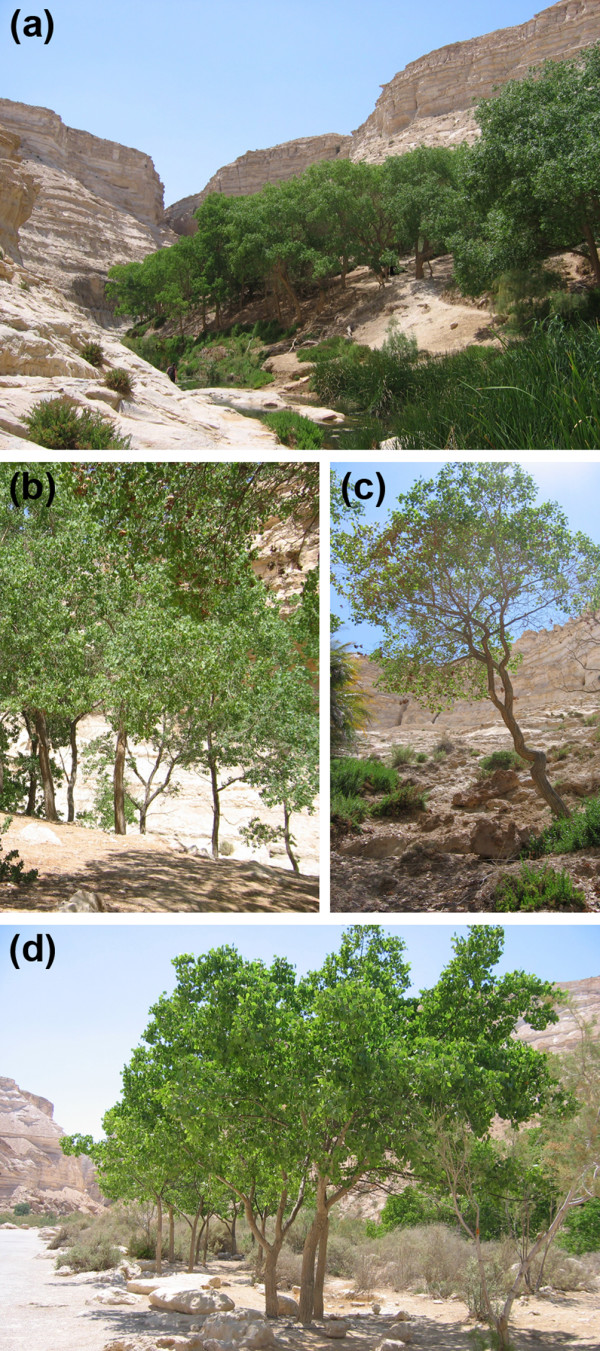
Ein Avdat areas A, B, C and P. **(a) **Area A trees grow near water and have a wide trunk and a large crown. **(b) **Trees from area B have narrower trunks than trees from area A. **(c) **Trees growing in area C on the slope, further from the water spring, have a narrow trunk and a small crown and display symptoms of being stressed. **(d) **Trees in area P, located 1 km from the Ein Avdat canyon, were planted in 1989 and originate from root suckers of trees growing in the valley. The trees at area P are irrigated once a week.

To capture as many as possible of the genes regulated by abiotic stress in *P. euphratica*, 17 different normalized and subtracted cDNA libraries were prepared from control and stress exposed trees. The treatments included several physiologically relevant abiotic stresses; salt, cold, drought, flooding and ozone (Additional data file 1). In addition, as a first step to elucidate the molecular mechanisms behind adaptation of *P. euphratica *to desert conditions, trees growing in the Ein Avdat valley were used for library construction (Figure 1). In total, 13,838 ESTs were obtained from the 17 libraries, with an average length of 478 nucleotides. The subtraction efficiency was estimated by 'electronic northern' analysis, that is, counting the abundance of various ESTs in different libraries (Additional data files 2 and 3). In the subtracted libraries prepared from leaves, all abundant photosynthesis genes were significantly removed (Additional data file 2) and in the subtracted libraries prepared from root tissues, genes with a putative role in abiotic stress were significantly enriched (Additional data file 3). The ESTs were submitted to GenBank with the accession numbers AJ767069 to AJ780913.

The 13,838 ESTs were analyzed and annotated within an openSputnik EST database as previously described [20]. Clustering and assembly of the EST sequences yielded 7,841 unigene features. These unigenes were exhaustively annotated for features including, but not restricted to, probable peptide sequence, Uniprot [21] matches, sequence overlap with complete and incomplete plant genome collections, and Gene Ontology assignments [22]. The entire database, including all annotative attributes and complete electronic northern analysis for all 17 EST libraries, can be viewed at the openSputnik website [23].

### Comparison to other plant genome and EST collections

The *P. euphratica *EST sequence collection was compared to sequences taxonomically oriented to the rest of the plant kingdom. The *P. euphratica *unigenes were anchored using BLAST methods to derive plant sequence datasets using an arbitrary expectation value of 1e-10. The datasets were constructed to represent both completed genomes (*P. trichocarpa*, *Arabidopsis thaliana *and *Oryza sativa*) and openSputnik unigene collections from clades of taxonomically related sequences. The numbers of sequences that could be matched to a sequence collection are shown in Table 1. The *P. euphratica *EST collection was highly similar to the *P. trichocarpa *genome assembly [24]; 97.6% of the ESTs can be found in the genome. Currently, approximately 250,000 ESTs from other *Populus *species are available from GenBank (Additional data file 4, dbEST release 062405). The *P. euphratica *EST collection overlaps these ESTs by 74%, which leaves approximately 2,000 novel ESTs in the present *P. euphratica *collection, indicating that the strategy of using normalized and subtracted stress enriched libraries has been successful at identifying novel *Populus *ESTs. Furthermore, the overlap of ESTs between *P. euphratica *and *P. tremuloides*, *P. trichocarpa*, *P. tremula *× *P. tremuloides*, *P. euramericana*, *P. alba *× *P. tremula*, *P. trichocarpa *× *P. deltoides*, *P. nigra*, *P. tremula *and *P. deltoides *varied between 21% and 63% (Additional data file 4). The coding content of *Populus *and *Arabidopsis *genomes has previously been shown to be highly similar [19]. Consistent with this, the *P. euphratica *EST collection also has a high overlap (69%) with the *Arabidopsis *genome. Interestingly, 763 sequences (approximately 10%) were only present in this *P. euphratica *EST collection and in the *P. trichocarpa *genome and not in any other plant EST or genome collections. This further validates the use of normalized and stress subtracted libraries for identifying novel ESTs. A total of 54 sequences with significant length and coding potential are not present in any other sequence collections, and may thus represent *P. euphratica *specific genes.

To estimate the representation of stress related ESTs in the *P. euphratica *collection, a comparison was also made to other EST collections or DNA microarray experiments performed with stressed plants (*P. euphratica*, *P. trichocarpa *× *P. deltoides*, *A. thaliana *and *Thellungiella halophila*) (Additional data file 5). Depending on the particular species or stress treatment, about 36% to 75% of known stress induced genes [6,17,25-27] have an equivalent EST in the *P. euphratica *EST collection (Additional data file 5).

### Functional annotation of the EST collection

*P. euphratica *unigene sequences were functionally and structurally annotated using Gene Ontology [22], and the complete assignments are summarized in Table 2 using the plant specific 'GOSlim' terms [28] under the three main categories of biological process, cellular component and molecular function. A broad range of functions and processes were represented in the *P. euphratica *ESTs. Annotation of the EST sequences with respect to GO terms for molecular function, biological process, and cellular component can be accessed at the openSputnik website [23].

### The *P. euphratica *DNA microarray

From the collection of 13,838 EST clones, a uni-gene set (selected using the CLOBB algorithm [29]; data not shown) was reamplified. In addition, 491 ESTs were amplified twice and are duplicated on the array. All PCR products were re-sequenced to verify the identity of the EST. The ESTs that could be postively identified through sequencing correspond to 7,342 distinct unigenes derived from the clustering of the sequences (HPT2 and CAP3 methods as described in [20]). To estimate the number of different genes on the array by allowing for the possibility of split unigenes, non-overlapping ESTs or unbridged assemblies, the unigenes were anchored into putative homologous groups using the available, but partial, *Populus *genome assembly. Using a stringent BLASTN expectation value of 1e-20, this identified 6,340 homologous groups. The data on the array may account for as many 6,340 distinct genes, and an undetermined number of paralogues.

### *P. euphratica *trees growing in the natural habitat of the species are exposed to salinity stress

The *P. euphratica *EST collection as described above contains several known abiotic stress regulated genes, but this is largely inferred from controlled laboratory experiments. To test which genes might be important for acclimation and adaptation to 'real' stress conditions, the *P. euphratica *microarray was used to further characterize trees grown in one of its natural habitats, the Ein Avdat valley, located in the Negev desert in Israel. In addition to measuring gene expression, we also analyzed leaves and soil for ionic content and the carbon stable isotope ratio δ^13^C/^12^C to estimate the level of salt and drought experienced by the trees.

We divided the valley into three different areas based on the distance from the small river running through it, distinct phenological differences of the trees growing on the canyon slope, as well as variations in the cambial activity and the width of the annual rings of the trees (Figure 1) [30]. Trees growing close to the river have a wide trunk and a large and healthy crown (area A). Trees in the transition area (area B) have narrower trunks than trees in area A, but large and healthy crowns. Trees growing on rocky, dry ground on the upper slope and far from the water source (area C) have a narrow trunk and a small crown and a very constrained growth. Control trees were selected at the Ein Avdat parking lot, 1 km away from the valley (area P), where the trees were periodically irrigated to provide optimal water supply (see Materials and methods). The trees at the parking lot, planted in 1989, originate from root suckers of trees growing in the valley. Leaves were sampled from at least nine individual trees in each area A, B and C, and from seven trees in area P. *P. euphratica *displays a foliar dimorphism and juvenile leaves differ largely from adults. In this case, only adult, heart shaped leaves where collected from the shaded and unshaded regions of the crown.

**Figure 2:**
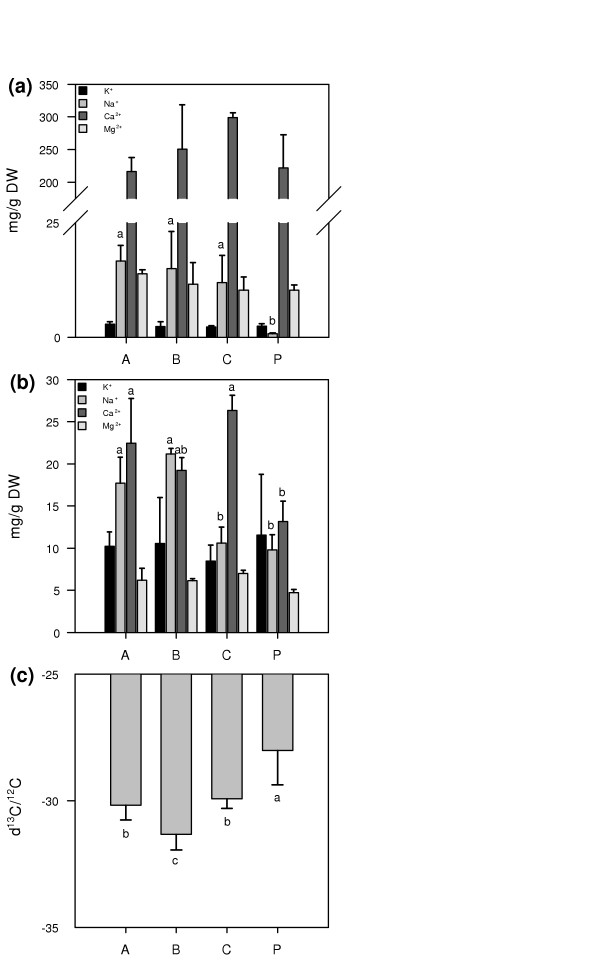
Estimation of salt and drought stress in area A, B, C, and P. Ion concentrations (mg/g dry weight (DW)) in **(a) **soil and **(b) **leaves taken from areas A, B, and C in the Ein Avdat valley and irrigated controls (area P). **(c) **^13^C content, expressed as δ^13^C (‰) was analyzed in the same samples used for ion concentration measurements. Significant (*p *≤ 0.05) differences are marked with different letters. The soil and leaf samples were harvested on 13 October 2004.

Soil samples for the analysis of ion content were collected beneath each tree (close to the trunk at approximately 10 cm depth) in the four different areas. Na^+ ^content was significantly higher (approximately 10 times) in the valley soil (areas A, B, and C) than in the soil from area P (Figure 2a). Other ions, including K^+^, did not differ significantly among the sites. *P. euphratica *trees growing in areas A and B accumulated more Na^+ ^in their leaves compared to trees from the irrigated area P (Figure 2b). Ca^2+ ^content was higher in leaves from areas A and C compared to those from area P.

An estimation of water use efficiency and thus the level of drought stress encountered by trees was obtained by measuring the carbon stable isotope ratio δ^13^C/^12^C (Figure 2c); δ^13^C/^12^C is a time-integrated index for the intrinsic water use efficiency, which is expected to increase during drought due to stomatal closure [31]. The trees from the irrigated area P were more water-use efficient (less negative δ^13^C/^12^C) than those in the valley. This suggests that the trees from the parking lot suffered more from water shortage than the trees in the valley (due to intermittent irrigation). An alternative and more likely interpretation, however, is that trees in the valley were exposed to a stress leading to strongly reduced carbon assimilation, in this case probably a combination of Na^+ ^and heat stress. In conclusion, trees from Ein Avdat areas A, B and C were exposed to salt and heat stress, but apparently not to drought stress.

### Gene expression in the Ein Avdat valley trees

**Figure 3:**
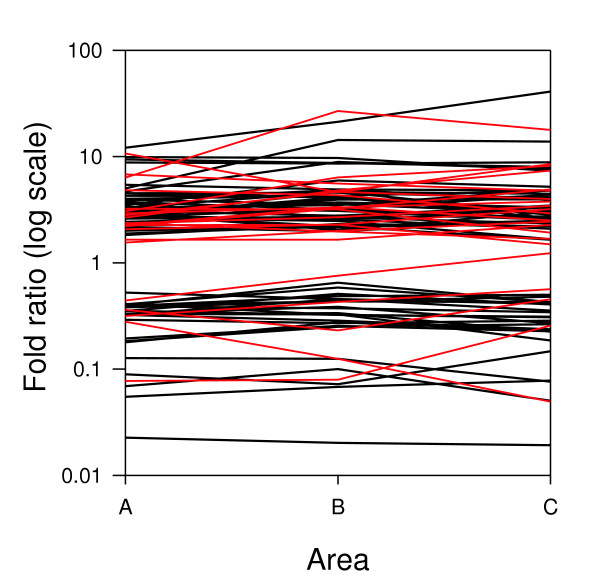
Transcript profiling of trees grown in area A, B, C and P.

For the microarray analysis, RNA was extracted from each of the nine individual trees sampled in areas A, B and C. Before labeling, RNA was pooled from three trees to give three biological repeats for each area. The same control RNA was used in all hybridizations and was prepared from a pool of leaves from seven trees growing at the parking lot (area P). Genes, grouped into functional categories, that consistently displayed higher or lower transcript levels in area A, B or C when compared to area P are shown in Additional data file 7 and are displayed graphically in Figure 3. Despite the highly different phenotypes of the trees in each area, their gene expression profiles were in most cases almost identical. Welch ANOVA analysis indicates that transcript levels for 22 genes (26%) showed significant differences between areas A, B and/or C, usually being either higher or lower in area C (indicated with an asterisk in Additional data file 7 and marked in red in Figure 3). About one third of the genes with higher transcript levels in area A, B and C have a putative role in response to abiotic stress. These include an aldehyde dehydrogenase and metallothioneins with putative roles in defense against oxidative stress. A plastid terminal oxidase that may play a role in photo-oxidative stress also had increased transcript levels.

In contrast to the relatively large number of genes with a putative role in defense against abiotic stress, no genes encoding transcription factors or other components of transcriptional regulation showed altered expression, although 153 ESTs on the array have the GO annotation 'regulation of transcription'. Consistent with this, only a few genes with a putative role in signal transduction (receptor-like Ser/Thr kinase, cyclic nucleotide and calmodulin-regulated ion channels and a phospholipase C) had increased transcript levels (Additional data file 7). As the salt stress imposed on the trees in the Ein Avdat valley is of a constitutive nature, the transcript level profiles detected in the microarray analysis are likely to be the end result of long term acclimation to saline desert conditions. Thus, genes responding rapidly after a stress treatment (including transcription factors) might be expected to be absent from the gene expression profile.

The transcript levels of several aquaporin water channels have been shown to be reduced during drought stress and to a smaller extent during salt stress [32]. This may help in protection against water loss under abiotic stress conditions [33]. In areas A, B and C, two aquaporins had decreased transcript levels. Other genes with lower transcript levels included those encoding chlorophyll a/b-binding proteins, chalcone synthase and dehydration-responsive protein RD22.

### Quantitative real time RT-PCR analysis

Seven ESTs representing genes with higher or lower transcript levels in the valley-grown trees than in area P trees, and spots with high and low hybridization intensities were used for quantitative real time RT-PCR (qPCR). An EST (AJ775831) encoding glucosidase II alpha subunit, which had displayed a constitutive expression level in all analyses performed with the *P. euphratica *DNA microarray, was used as an internal control to which gene expression was normalized (ΔC_T_). The same RNA samples from areas A, B, and C and the area P control that were used in the DNA microarray analysis were used as templates in qPCR (Table 3). For most of the genes, transcript fold ratios were close to the microarray results in both analyses, indicating the reliability of the *P. euphratica *DNA microarray. One gene (AJ780552) displayed a higher fold-induction in the PCR analysis, which probably reflects the higher dynamic range of qPCR compared to array analysis [34]. For only one gene (AJ769631) where the DNA microarray data implied a minor change in expression levels did the qPCR not show the same differential expression.

### Metabolic profiling

The accumulation of 22 different metabolites in leaves of *P. euphratica *trees was examined by gas chromatography coupled to mass spectrometry (GC-MS). The metabolites were analyzed initially in the leaves used also for the transcriptome analysis in areas A, B and C but not in P. The analysis was repeated during the following year for areas A, B, C and P. We present data from this last sampling as metabolite contents in areas A, B, and C did not change with sampling dates. Metabolite profiles of leaf extracts from six individual plants from each area (A, B, C, and P) were compared (Table 4). Trees from area A (which accumulated more Na^+^; Figure 2a) displayed significantly higher levels of the amino acids β-alanine, valine and proline than trees from other areas, including the less saline area P. Proline has previously been shown to accumulate to high levels in Na^+ ^or osmotically stressed *P. euphratica *[35]. Likewise, glycerol, glyceric acid, and myo-inositol had also significantly higher concentrations in trees from area A compared to trees from the other areas. Fructose and mannitol, on the contrary, were detected in significantly lower concentrations in trees from the area A. No significant and/or clear differences between the trees from different areas were detected in any of the other metabolites analyzed.

For one of the metabolites detected, galactinol, the corresponding biosynthesis gene (galactinol synthase) had approximately threefold higher transcript levels in areas A, B and C (Additional data file 7). Galactinol itself did not display any significant increase in the leaves. Nevertheless, galactinol is a precursor of raffinose, which showed significant increases in areas B and C (Table 4). Significant changes were also detected in myo-inositol, which is a precursor of galactinol [36].

## Discussion

### *P. euphratica *trees growing in the Ein Avdat valley are exposed to Na+ stress

Soil in the Ein Avdat valley contains high concentrations of Na^+ ^(Figure 2a). This is also reflected in the vegetation composition in the valley, which includes several halophytic species (*Juncus maritimus *Lam, *Suaeda vera*, *Atriplex halimus*, *Imperata cylindrical*, *Limonium pruinosum *and *Zygophyllum dumosum *Boiss.). Maintenance of cellular ion homeostasis is important for vital activities in all organisms. High salinity, in addition to osmotic stress, causes ion imbalances and consequently cell death. K^+ ^is one of the major cations in the cytosol and is responsible for the maintenance of membrane potential. Under normal growth conditions, K^+ ^concentration in plant cells is higher than Na^+ ^concentration. High Na^+ ^in soil causes inhibition of K^+ ^uptake, resulting in a severe increment in the Na^+^/K^+ ^ratio, which is detrimental for photosynthetic activity and causes inhibition of growth and ultimately cell death [37]. *P. euphratica *trees grown in highly saline soils (Figure 2a) accumulated high Na^+ ^concentration in leaves (approximately twofold more Na^+ ^than K^+^; Figure 2b) and still continued growing without visible symptoms of severe stress. This clearly indicates that *P. euphratica *has developed an efficient mechanism to tolerate high salt levels. Accordingly, *in vitro *exposure of *P. euphratica *to salt showed that Na^+ ^is preferentially accumulated in the cell wall, which thus protects the cytosol from Na^+ ^toxicity [38]. The carbon isotope ratio δ^13^C/^12^C revealed that the trees were likely not suffering from severe drought stress (Figure 2c). This may be due to the fact that *P. euphratica *trees produce a deep and extensive root system, giving the trees access to deep water even when they were far from the river (area C) [9,39].

### The *P. euphratica *EST collection contains stress related ESTs

Survival and growth of *P. euphratica *trees under the unfavorable conditions of high soil salinity and heat stress suggest that they are well adapted to the local desert conditions. Thus, sequencing of cDNA libraries in combination with DNA microarray analysis was used to elucidate the mechanisms underlying this adaptation. Construction of the cDNA libraries was based on maximizing the number of sequences relevant to stress tolerance and adaptation, in particular salt stress. To reach this goal, normalized libraries from Ein Avdat valley-grown adult trees and subtracted libraries from NaCl and several other abiotic stress-treated juvenile trees were constructed (Additional data file 1).

The EST collection obtained was compared to other poplar stress EST collections and salt DNA microarray experiments to estimate the amount of stress related genes obtained (Additional data file 5). Gu *et al*. [6] used a similar strategy and employed the suppression subtractive hybridization (SSH) method to identify genes upregulated by salt in *P. euphratica*. Of the 58 sequences reported, 43 (74%) were present in our EST collection. Christopher *et al*. [17] sequenced 928 ESTs from systemically wounded leaves of hybrid poplar (*P. trichocarpa *× *P. deltoides*) and performed a macroarray analysis on systemically wounded leaves. Of the genes listed as having probable or potential herbivory or wounding functions, 20 of 33 sequences (60%) were represented in our EST collection. In the macroarray analysis, they identified 18 genes with more than a tenfold increased transcript level in response to wounding; 12 of these 18 sequences (67%) were present in our EST collection. In our collection, the majority of highly wound-induced genes were obtained from the normalized control libraries, indicating that *P. euphratica *trees may constitutively express defense genes. Whether genes that could contribute to salt tolerance of *P. euphratica *also are constitutively expressed remains to be determined. Wounding- and poplar mosaic virus-regulated genes have also been identified in *P. trichocarpa *× *P. deltoides *using microarray analysis [25]. Of the 150 genes induced twofold or more by virus and/or wounding, 113 (75%) were present in the *P. euphratica *EST collection (Additional data file 5), indicating that even though all cDNA libraries were constructed from trees exposed to abiotic stress, there were many ESTs related to biotic stress present in the *P. euphratica *EST collection.

As only three *Populus *array experiments relating to stress have been published, a comparison was also made to transcript profiling of salt stressed *A. thaliana*. Seki *et al*. [26] analyzed 7,000 genes in *A. thaliana *and found that 227 genes displayed more than fivefold increased transcript levels and 103 genes decreased levels in response to the NaCl treatment. A comparison of these genes with the *P. euphratica *EST collection showed that 81 (36%) of the *Arabidopsis *genes with increased transcript levels in response to salt stress have a direct match in our EST collection (Additional data file 5). Among the genes with reduced transcript levels, 56 (54%) were present in our EST collection. Not surprisingly, the *P. euphratica *genes with a match to the Na-regulated *Arabidopsis *genes were predominantly from the normalized Ein Avdat library and the subtracted libraries, whereas the down regulated genes mostly had matches in the control libraries. Overall, this comparison with known stress regulated genes indicates that a wide collection of stress related genes was obtained in the *P. euphratica *EST collection.

### Transcript profiling of Ein Avdat trees

A uni-gene set of the *P. euphratica *EST collection was printed on microarrays, which were used to profile gene expression in leaves harvested from Ein Avdat areas A, B and C. Leaves from trees growing in the least saline soil (area P) were used as controls. In *A. thaliana*, typically 5% to 30% of the genes displayed changed levels of transcripts in response to various abiotic stress treatments [26,40]. In striking contrast, the number of *P. euphratica *genes that displayed different transcript levels expressed in the Ein Avdat experiment was only approximately 1% of the genes on the array. Furthermore, the trees in areas A, B and C had similar transcript profiles (Additional data file 7). For some genes, for example an early light-induced protein, there was a significantly higher expression in area C compared to the other areas (Additional data file 7). These genes with higher expression in area C might be important for adaptation to the highly stressful conditions on the dry, rocky slope (Figure 1). The majority of the genes with altered transcript levels belong to only a few functional categories: 'protein metabolism', 'response to abiotic or biotic stimulus', 'metabolism' and 'biological process unknown'. One reason for the relatively low number of genes with different transcript levels could be that the Ein Avdat trees have been growing in the valley for decades and are well acclimated to these growth conditions, and thus may have their defense mechanisms fully activated. This could also explain why so relatively few genes involved in signal transduction and no genes involved in control of transcription were identified as displaying different transcript levels. A systematic genotyping of the Ein Avdat trees with Simple Sequence Repeat (SSR) markers did not detect any polymorphism among the trees, suggesting that the *P. euphratica *trees are ramets of the same genotype, probably propagated through root suckers (I Beritognolo and R Muleo, personal communication; see also [39,41]). Thus, the trees at each area may be expected to respond similarly to stress conditions, which also might explain their almost identical transcript profiles (Additional data file 7).

Interestingly, some of the genes with increased transcript levels in areas A, B, and C (galactinol synthase, aldehyde dehydrogenase, β-amylase, ferritin and cysteine protease) have previously been shown to be upregulated in *Arabidopsis *during combined heat and drought stress [42]. The galactinol synthase gene was only found in the Ein Avdat EST library, indicating that galactinol (or raffinose as indicated by the metabolite profiling; Table 4) might be used as a compatible solute during salt stress. Galactinol synthase is also one of the genes with increased transcript levels in controlled laboratory experiments with salt exposed *P. euphratica *[38]. Abiotic stress causes the formation of reactive oxygen species that can react further with cellular components such as lipids and proteins. None of the 'classic' enzymes involved in antioxidant defense, including catalase, ascorbate peroxidase and superoxide dismutase, showed altered transcript levels. However, two other proteins, aldehyde dehydrogenase and metallothioneins, might fulfill a similar role in antioxidant defense. A result of oxidative damage is the formation of reactive and toxic aldehydes. In *A. thaliana*, an aldehyde dehydrogenase (*AthALDH3*) gene was induced by salt stress and other abiotic stresses [43]. Transgenic plants overexpressing *AthALDH3 *displayed increased tolerance to salt and dehydration stress and accumulated less reactive aldehydes derived from lipid peroxidation [43]. The increased transcript abundance of an aldehyde dehydrogenase in *P. euphratica *suggests that it could use this defense strategy to reduce damaging effects from oxidative stress. Metallothioneins are low molecular mass cysteine-rich proteins that can bind heavy metals and have a proposed role in detoxification of heavy metals. Expression of poplar metallothioneins in a cadmium sensitive yeast strain conferred cadmium tolerance [44]. The transcript levels of poplar metallothioneins increase during senescence, heavy metal treatment and virus infection [13,25,44]. The presence of several cysteines in the metallothioneins suggests that they might be involved in the detoxification of reactive oxygen species or in maintenance of redox levels. Recently, recombinant rice and watermelon metallothinoneins have been shown to possess high superoxide and hydroxyl radical scavenging capacity [45,46]. Metallothionein ESTs were highly abundant in Ein Avdat and control libraries (Additional data file 2). Furthermore, the microarray analyses indicated that they were more highly expressed in trees growing in areas A, B and C than in trees growing in the less saline area P (Additional data file 7). This suggests that metallothioneins might play a role in detoxification of reactive oxygen species in stressed trees.

The genes with highest fold-induction in transcript levels in the Ein Avdat valley encode cysteine proteases. Cysteine proteases have previously been shown to be induced under both salt and drought stress [47]. Their role could be to enhance degradation of stress-denatured proteins. Alternatively, elevated cysteine protease expression is frequently observed during senescence in *Populus *[13,15]. As the samples for array analysis were harvested late in autumn, it is possible that the trees in the valley (particularly in area C) might develop senescence faster than the trees at the parking lot, and this might lead to elevated cysteine protease expression.

Recently, the halophyte salt cress (*T. halophila*) has been established as a model for research on salt tolerance due to its high sequence identity with *A. thaliana *[48,49]. Comparative microarray experiments performed on *A. thaliana *and *T. halophila *with the same NaCl treatment revealed that the salt sensitive *A. thaliana *responded to the stress by modified expression of a relatively large number of genes, while the expression of only a few genes (six times less) changed in the tolerant *T. halophila*. Furthermore, direct hybridization of control RNA from both species on the same array showed that the tolerant species over-expressed several stress-related genes under non-stressed conditions [49]. Thus, the salt tolerant species constitutively expresses defense genes and does not show further induction of genes upon stress treatment. Forty-nine percent of the stress related genes constitutively expressed in *T. halophila *were present in the *P. euphratica *EST collection (Additional data file 5). *P. euphratica *resembles *T. halophila *in several ways; both are tolerant to salt stress and display constitutive expression of genes related to, and induced by, biotic stress. Further support for the hypothesis that *P. euphratica *constitutively expresses defense genes and responds with few changes in gene expression following stress comes from our preliminary array experiments using salt stressed *P. euphratica *and *P. tremula *(which is salt sensitive); *P. tremula *displayed 10 times more genes with modified transcript levels than *P. euphratica *(B Vinocur, M Brosché, J Kangasjärvi, A Altman, unpublished data). To be fully confirmed, this hypothesis requires additional study, especially direct microarray hybridization comparisons between *P. euphratica *and stress sensitive poplars such as *P. tremula *or *P × canescens*.

### Metabolic profiling of Ein Avdat trees

To complement the transcript profiling we also analyzed the accumulation of 22 metabolites in the leaves collected from the experimental sites. Increased concentration of several amino acids such as β-alanine, valine and proline was observed, particularly in area A (Table 4). Rizhsky *et al*. [42] reported similar increases in different amino acids such as isoleucine, leucine, valine, β-alanine and tyrosine as a response to heat and drought stress, and proline, cysteine and serine have increased concentrations following cold stress [50]. Among the amino acids analyzed, β-alanine had the highest accumulation in *P. euphratica *leaves from areas A and B. β-Alanine is the precursor of β-alanine-betaine, a quaternary ammonium compound that has similar osmoprotective function as glycine-betaine and accumulates in species belonging to the highly salt tolerant family *Plumbaginaceae *[51]. Therefore, it is possible that amino acid accumulation supports increased production of metabolites that are part of a defense under very saline conditions.

The concentration of several metabolites such as fructose, glycerol, malate, maltose, mannose, galactinol, myo-inositol, putrescine, raffinose, sucrose, and trehalose increase dramatically in response to heat, drought and cold stress in *Arabidopsis *[42,50]. In *P. euphratica*, however, the changes in stress responsive carbohydrates and organic acids were of limited extent at the four different field sites. Considering the high accumulation of Na^+ ^in field-grown leaves (Figure 2b), as well as in controlled salt experiments [38], it is possible that sodium itself can act as an osmolyte as in halophytes.

## Conclusion

*P. euphratica *trees growing in the Ein Avdat valley are naturally exposed to high salinity, as well as to heat stress. Despite this, most of the trees did not display severe symptoms of stress (except for the stunted phenotype of trees growing in the driest area), and from a molecular perspective, relatively few changes occurred at the transcript and metabolite levels. It is possible that mechanisms not studied here (for instance, Na^+ ^transporter activity) may contribute to the tolerance and adaptation to the high salinity encountered in this population. The EST collection and microarray described here represent excellent tools for further molecular research into the strategies used by *P. euphratica *to grow in desert conditions.

## Materials and methods

### Plant material

The Ein Avdat valley is located in the Negev desert in Israel at 450 m above sea level and a latitude of 30° 49' 30" N, and a longitude of 34° 46' 0" E. The average rainfall is 100 to 200 mm per year with most of the rain falling during winter time. Adult heart shaped leaves were collected from trees in areas A, B, and C for the cDNA library during the summer seasons of 2000 to 2001. Material was also harvested from young *in vitro *plantlets of a clone (clone B2) derived from a single tree from area B. Samples for microarray analysis were taken from areas A, B, and C and from the Ein Avdat parking lot (area P, 1 km from the valley) on 27 November 2003, and for ion, isotope and metabolite profiling on 27 November 2003 and 13 October 2004. The parking lot trees were irrigated with non saline tap water for 24 hours once a week.

For normalized control libraries, leaves, shoots and roots were harvested from 8 month old *P. euphratica *seedlings. Seeds were obtained from the Forest Administration Division, Xinjiang Corps of Production and Construction, Urumqi, PR China. The seeds were sown on sand that had a high content of clay (6%) and germinated at 12 hours light with 250 μmol m^-2 ^s^-1 ^photosynthetically active radiation, 25°C, and a relative air humidity of 60% in a climate chamber (Weiss, Giessen, Germany). The seedlings were potted into soil (Fruhstorfer Erde N, Archut, Germany), transferred to a greenhouse with natural light and watered daily with Long Ashton macronutrient solution [52].

For stress treatments, leaves and roots were harvested from *P. euphratica *subjected to the following treatments: elevated CO_2 _(plants submitted to an atmosphere of 700 ppm for 60 days); different irradiance levels (plants under three different irradiance regimes corresponding to 48%, 18% and 8% external irradiance) for 60 days; drought stress (plants submitted to a 21 day cycle of drought with 5% to 3% volumetric soil humidity) before harvesting; flooding stress (plants submitted during 21 days to a complete submergence of the root system); ozone (plants submitted to 200 nl l^-1 ^of O_3 _for 7 and 20 h); cold and freezing (leaves harvested from plants acclimated at 5 days of +2°C and from plants exposed to two and seven days of -2°C following the acclimation period); salt stress (leaves and xylogenic cambium harvested from plants exposed to 150 mM NaCl for 0.5 h, 1 h, 1.5 h, 2 h, 5 h, 10 h, 24 h and 48 h and roots harvested after 40 minutes, 1.5 h, 8 h and 20 h); cadmium stress (plants were stressed with 50 μM CdCl_2 _and roots harvested after 12, 24 and 48 h).

### Ion and isotope measurements

Leaves were frozen in liquid nitrogen and freeze dried for two days. Dry powder samples (250 mg) were then extracted with 5 ml 65% HNO_3 _at room temperature overnight to release the free ions and then digested for 2 h at 180°C. The supernatants were diluted in deionized water and ion analysis was carried out using an ICP-AES Spectroflame (Spectro Analytical Instruments, Kleve, Germany). The data were analyzed with the Tukey multiple comparison algorithm (*p *= 0.05). Soil samples were dried at 70°C and subsequently digested with a nitric acid pressure system according to Heinrichs *et al*. [53]. Quantification of elements was carried out by ICP-OES (inductively coupled plasma-optical emission spectrometry; Spectro Analytical Instruments) at l = 559 nm. One way ANOVA tests were performed with the Tukey honestly significant difference (HSD) *post hoc *test. For isotope measurements freeze-dried leaves were finely ground using a mill (CB2200, CEP Industrie, Dept Sodemi, Saint-Ouen l'Aumône, France). ^13^C content in bulk leaf tissue was measured with an isotope mass spectrometer (Finnigan Mat; Delta S, Bremen, Germany). ^13^C content was expressed as the ^13^C/^12^C ratio relative to the Pee Dee Belemnite standard as a per mil deviation from this standard. The precision of the spectrometric analysis (standard deviation in δ) of the laboratory standard (ground leaves of *Quercus *ssp.) was 0.15‰ (n = 201).

### Construction of cDNA libraries

RNA was isolated from *P. euphratica *leaves or roots using the method described in Chang *et al*. [54]. Total RNA from root samples was DNase I treated before mRNA isolation. mRNA was isolated using oligo-tex beads (Qiagen, Hilden, Germany). Normalization of mRNA populations was performed as described in Sasaki *et al*. [55]. Normalized cDNA libraries were constructed from mRNA using the SuperScript plasmid system with Gateway technology for cDNA synthesis and cloning (Invitrogen, Carlsbad, CA, USA). Subtracted libraries were constructed by two different methods. First by using the PCR select cDNA subtraction kit (BD Bioscience Clontech, Palo Alto, CA, USA; also called SSH [56]), with the resulting PCR products cloned into pGEMT-Easy T-vector (Promega, Madison, WI, USA). The second subtraction method used restriction enzyme digestion with cap-selection. Tester cDNA was prepared by synthesizing cDNA from 500 ng mRNA with an oligo-dT adaptor primer (5' GACTAGTTCTAGATCGCGAGCGGCCGCCCTTTTTTTTTTTTTTTTVN3') in the presence of a 'cap select' primer (5'AAGCAGTGGTATCAACGCAGAGTGTCGACGGG3'), which binds to the end of a newly synthesized cDNA (see [57] for a description of the CapSelect technique). A driver was prepared by single-strand PCR amplification from control libraries and by double-stranded cDNA synthesis from control mRNA. The driver was then hybridized to the tester in a ratio of at least 50:1 for 24 to 48 h. After hybridization, double stranded molecules (representing common transcripts) were degraded by digestion with a panel of restriction enzymes (*Rsa*I, *Nla*III, *Bfa*I, *Sau3*AI; New England Biolabs, Beverly, MA, USA). The remaining undigested single-stranded cDNAs were size selected and subsequently amplified by PCR using primers designed for the 5'cap and 3' poly-A tail (5'AAGCAGTGGTATCAACGCAGAGT3' and 5'GACTAGTTCTAGATCGCGAGC3'). The resulting PCR products were digested with *Not*I and *Sal*I, size selected, and cloned into pSPORT1 (Invitrogen, Carlsbad, CA, USA). The subtracted libraries were subjected to a stringent quality control to determine how many clones from each library to sequence. Two parameters were tested, 'subtraction efficiency' and 'complexity'. Essentially, subtraction efficiency was judged by assessing the removal of abundant control cDNAs. Complexity was judged by determining the percentage of different cDNAs in the library. To test this, 96 clones were sequenced from each library. To pass 'subtraction efficiency', a maximum of one of the obtained sequences should correspond to any of the five most abundant cDNAs from the normalized libraries (rbcS, lhcb, ferredoxin, metallothionein, PSII 10 kDa protein). Furthermore, at least 70% of the sequences had to be sequences not already present from the normalized libraries. To pass 'complexity', the number of independent sequences had to be at least 60%. The following libraries passed the quality control: P8, P9, P10, P11, P15, P16 and P17.

### EST sequencing and annotation

EST sequencing was performed as described in Laitinen *et al*. [58]. The sequences have GenBank accession numbers AJ767069 to AJ780913. All the sequences were quality checked before clustering and integration into a Sputnik EST database as described in [23,58]. Unigene sequences are assigned a unique id based on their 5'-most EST sequence. A prefix 'C_' denotes that the unigene represents a multi-member 'cluster' of sequences. Singleton sequences are prefixed with 'S_'. Peptide sequences were derived for all unigenes using the ESTScan application [59]. Prior to the ESTScan predictions, a *P. euphratica *specific ESTScan model was created by training with open reading frames identified through BLASTX of the unigenes against the Swissprot database with results filtered using the expectation value of 1e-10. The peptide predictions were used to estimate the amount of protein coding sequence (CDS) within both individual EST and cluster consensus sequences. Sequences were annotated for homology using the BLASTN and BLASTX algorithms against a non-redundant protein sequence database, the Swissprot database, the *A. thaliana *and *O. sativa *genome databases, and sequence databases containing the aggregated openSputnik consensus sequences for Asterid, Eurosid, Caryophyllid, and monocot genomes. Sequences were additionally functionally characterized in context with the MIPS Funcat and Gene Ontology GO terms [28]. GO terms were derived using two independent methods: one using the Swissprot to Gene Ontology mappings, the other using the InterPro to Gene Ontology mappings.

### Construction of microarrays

The ESTs were clustered using the CLOBB algorithm and based on the clusters, a uni-gene set were selected for reamplification as previously described [29,58]. All PCR-products were sequenced to verify correct identity. The Populus microarray contains 7,662 different populus ESTs (of which 491 ESTs were amplified twice and are duplicated on the array, for a total number of 8,153 populus ESTs; these ESTs correspond to 7,342 distinct unigenes derived from the clustering of the sequences (HPT2 and CAP3), and spiking and negative controls that were amplified from the *Arabidopsis *functional genomics consortium control set [60] and from additional *Caenorhabditis elegans *clones. The number of genes corresponding to the arrayed ESTs was estimated by anchoring ESTs to the available Poplar genome scaffold using the BLASTN method, with results filtered at 1e-20. Sequences were merged if they overlapped or appeared within the same 5 kbp interval on the assembled genome scaffold.

For printing, 5 μL of purified fragments were transferred to a 384 printing plate together with 5 μL of 6 × SSC solution. The ESTs were printed onto poly-lysine coated slides with a Genemachines OmniGrid 100 (Genomic Solutions, Ann Arbor, MI, USA) using 16 SMP3 pins (TeleChem International, Sunnyvale, CA, USA). Printing conditions were 48% to 50% humidity and 21°C.

### Probe labeling and hybridization

Three biological repeats were used from each of the areas (A, B and C; Figure 1) in the Ein Avdat valley; each of the independent biological repeats contain leaves from two or three trees. The parking lot (area P) control sample was a pool of leaves from seven trees. To avoid bias in the microarray evaluation as a consequence of dye-related differences in labeling efficiency and/or differences in recording fluorescence signals, dye labeling for each paired sample (Area/Parking lot control) was reversed in two individual hybridizations. Thus, a total of six hybridizations per area were obtained. The complete protocols for probe labeling and hybridization, normalized data and raw data files are available from the ArrayExpress database [61] under the accession E-MEXP-182.

### Microarray data analysis

Images were analyzed in GenePixPro 5.1 (Axon Instruments, Union City, CA, USA). Visually bad spots or areas on the array and low intensity spots were excluded. Low intensity spots were determined as spots where less than 55% of the pixels had intensity above the background + 1SD in either channel. Using these criteria, 31% to 58% of the spots gave a positive signal from the 18 hybridizations. The data from GenePixPro 5.1 were imported into GeneSpring 7 (Silicon Genetics, Redwood City, CA, USA) and normalized with the Lowess method. The background subtracted median intensities were used for calculations. Up- and downregulated genes were selected using two criteria: the gene should consistently have twofold increased or decreased transcript levels in all biological repeats, there should be a signal from the spot from at least four of six hybridizations for each area, and the gene induction or repression should be statistically significantly different from a ratio of 1.0 determined with Students *t* test in GeneSpring. Gene expression in areas A, B, and C was compared using Welch ANOVA assuming that groups have unequal variances. After Benjamini and Hochberg false discovery rate correction for multiple testing, a false discovery rate of 0.05 or less was considered statistically significant. Tukey's *post-hoc *test was applied to identify the areas that differ from each other.

### Quantitative real time RT-PCR

The microarray results were verified with qPCR. Reverse transcription was performed with 6 μg of DNase I treated total RNA with SuperScript III according to the manufacturer's instructions (Invitrogen). The reverse transcription reaction was diluted to a final volume of 100 μl, and 1 μl was used as template for the PCR using SYBRgreen PCR master mix (Applied Biosystems, Foster City, CA). PCR was performed in duplicate using ABI Prism 7000 default cycling conditions (Applied Biosystems). The primers used for PCR are listed in Additional data file 6. The raw threshold cycle (Ct) values were normalized against glucosidase II alpha subunit (shown to have a constant expression in all experiments performed on the *P. euphratica *DNA microarray) to obtain normalized ΔCt values that were then used to calculate the difference in expression levels between areas A, B and C and the parking lot control.

### Metabolite profiling: GC-MS analysis

The extraction protocol was modified from Roessner-Tunali *et al*. [62] and Schauer *et al*. [63]. Briefly, frozen leaf tissue powder (100 mg dry weight) was extracted in 1.4 ml of 80% (v/v) aqueous methanol in a 2.0 ml microcentrifuge tube. Ribitol (120 μl of 0.2 mg ml^-1 ^water) was added as an internal standard prior to incubation. The mixture was extracted for 15 minutes at 70°C under 200 rpm. The extract was vigorously mixed with 1,500 μl water and 750 μl chloroform andsubsequently centrifuged 15 minutes at 4,000 rpm. Aliquots of the methanol/water supernatant (150 μl) were dried in vacuum overnight. The dry residue was modified for GC-MS analysis according to Schauer *et al*. [63]. Residues after reduction were redissolved and derivatized for 90 minutes at 37°C in 60 μl of 30 mg ml^-1 ^methoxyamine hydrochloride in pyridine followed by a 30 minute treatment with 120 μl of N-methyl-N-[trimethylsilyl]trifluoroacetamide at 37°C. A retention time standard mixture (10 μl of 0.029% (v/v) n-dodecane, n-pentadecane, n-nonadecane, n-docosane, n-octacosane, n-dotracontane, and n-hexatriacontane dissolved in pyridin) was added before trimethylsilylation. Sample volumes of 1 μl were then injected onto the GC column on a splitless mode.

The GC-MS system was run as previously described [63] with some modifications. It was composed of a Pal autosampler (Ophir analytic Tel Aviv, Israel, representative of CTC analytic, Zwingen, Switzerland), a TRACE GC 2000 gas chromatograph, and a TRACE DSQ quadrupole mass spectrometer (Restek, Bargal Analytics, Tel Aviv, Israel, representative of ThermoFinnigan, Hemel Hempstead, UK). GC was performed on a 30-m Rtx_5Sil MS column with 0.25 μm film thickness (ThermoFinnigan). The injection temperature was set at 297°C, the interface at 280°C, and the ion source adjusted to 200°C. Helium was used as the carrier gas at a flow rate of 1 ml min^-1^. The analysis was performed under the following temperature program: 5 minutes of isothermal heating at 70°C, followed by a 5°C per minute oven temperature ramp to 350°C, and a final 5 minute heating at 330°C. Mass spectra were recorded at 2 scan sec^-1 ^with a scanning range of 40 to 600 m/z. Both chromatograms and mass spectra were evaluated using the XCALIBUR v1.3 program (ThermoFinnigan). A retention time and mass spectral library for automatic peak quantification of metabolite derivatives were implemented within the NIST 2.0 method format. Substances were identified by comparison with authentic standards, as described in Roessner-Tunali *et al*. [62].

The levels of the compounds were calculated as the relative response ratio of peak areas of different compounds related to the peak area of ribitol (which served as an internal standard), and normalized with respect to the dry weight of the sample (according to Nikiforova *et al*. [64]). Data are presented as the average of six separate measurements and analyzed statistically with the Tukey multiple comparison algorithm (*p *= 0.05) incorporated into JMP statistical software (JMP, Statistics and Graphics Guide: Version 3, SAS Institute Inc. Cary, NC, USA).

## Additional data files

The following additional data are available with the online version of this paper. Additional data file 1 is a description of the cDNA libraries used for EST sequencing. Additional data file 2 lists data from the electronic northern analysis showing the most abundant ESTs in leaf libraries. Additional data file 3 lists data from the electronic northern analysis showing the most abundant ESTs in root libraries.Additional data file 4 is a comparison of the *P. euphratica *EST collection with ESTs from other *Populus *species. Additional data file 5 is a comparison of the *P. euphratica *EST collection with other stress enriched EST collections or microarray experiments. Additional data file 6 list the primers used for real time quantitative RT-PCR. Additional data file 7 shows the microarray analysis of leaf samples taken from areas A, B, C and P in the Ein Avdat valley.

## Supplementary Material

Additional data file 1 A description of the cDNA libraries used for EST sequencing. Click here for file

Additional data file 2 Data from the electronic northern analysis showing the most abundant ESTs in leaf libraries. Click here for file

Additional data file 3 Data from the electronic northern analysis showing the most abundant ESTs in root libraries. Click here for file

Additional data file 4 A comparison of the *P. euphratica *EST collection with ESTs from other *Populus *species. Click here for file

Additional data file 5 A comparison of the *P. euphratica *EST collection with other stress enriched EST collections or microarray experiments. Click here for file

Additional data file 6 The primers used for real time quantitative RT-PCR. Click here for file

Additional data file 7 Microarray analysis of leaf samples taken from areas A, B, C and P in the Ein Avdat valley. Click here for file

## Figures and Tables

**Table 1:** Comparison of the *P. euphratica *unigene collection with other sequence collections from whole genomes or EST projects

Sequence collection	Matches	Unique	
All			**7,841**

*Populus *genome	7,671	763	
*Arabidopsis *genome	5,434	2	
Rice genome	1,562	0	
*Populus *EST sequence	5,780	5	
Rosid EST sequence	4,597	1	
Asterid EST sequence	3,490	4	
Caryophyllid EST sequence	2,081	0	
Monocot sequence	2,135	3	
GenBank sequence	5,495	0	
Short sequences	275	20	
Low protein coding potential	728	28	
**Remainder**			**54**

All *P. euphratica *unigenes were compared against reference sequence collections to investigate sequence overlap and to identify the number of sequences unique to this sequence collection. The reference sequence collections include the draft *Populus *genome, the *Arabidopsis thaliana *genome, the rice genomes and pooled collections of openSputnik EST collections representing large collections from species taxonomically assigned to the plant groups of rosid, asterid, caryophyllid and monocot. Also included in the reference sets are the sequences having a match to an annotated protein in the UniProt database or *P. euphratica *sequences that are either short (less than 100 nucleotides) or have a low protein coding potential (less than 25% protein coding). In the table, the reference sequence collection is displayed along with the number of *P. euphratica *sequences that can be matches to the reference sequence collection and the number of sequences that are unique to this sequence collection. All blast analyses were performed using an arbitrary expectation value of 1e-10. The remainder (54) represents the number of sequences that have no match within any of the challenge datasets and may thus represent *P. euphratica *specific genes.

**Table 2:** Gene Ontology annotation of *P. euphratica *unigene sequences

GO term	GO	Number of sequences
**biological_process**	GO:0008150	2,141
behavior	GO:0007610	48
cellular process	GO:0009987	681
cell communication	GO:0007154	167
cell-cell signaling	GO:0007267	1
response to extracellular stimulus	GO:0009991	11
signal transduction	GO:0007165	150
cell death	GO:0008219	33
cell differentiation	GO:0030154	87
cell growth and/or maintenance	GO:0008151	546
cell cycle	GO:0007049	83
cell growth	GO:0016049	5
cell organization and biogenesis	GO:0016043	157
cell homeostasis	GO:0019725	25
transport	GO:0006810	397
development	GO:0007275	211
cell differentiation	GO:0030154	87
embryonic development	GO:0009790	1
flower development	GO:0009908	12
morphogenesis	GO:0009653	96
regulation of gene expression, epigenetic	GO:0040029	11
reproduction	GO:0000003	60
ripening	GO:0009835	29
physiological process	GO:0007582	2,096
photosynthesis	GO:0015979	74
response to stress	GO:0006950	270
response to endogenous stimulus	GO:0009719	84
response to external stimulus	GO:0009605	229
response to abiotic stimulus	GO:0009628	69
response to biotic stimulus	GO:0009607	199
metabolism	GO:0008152	1,758
amino acid and derivative metabolism	GO:0006519	123
biosynthesis	GO:0009058	512
carbohydrate metabolism	GO:0005975	255
catabolism	GO:0009056	317
electron transport	GO:0006118	247
energy pathways	GO:0006091	154
lipid metabolism	GO:0006629	125
nucleobase\, nucleoside\, nucleotide and nucleic acid metabolism	GO:0006139	367
DNA metabolism	GO:0006259	123
transcription	GO:0006350	36
protein metabolism	GO:0019538	757
protein biosynthesis	GO:0006412	252
protein modification	GO:0006464	222
		
**cellular_component**	GO:0005575	33
cell	GO:0005623	7
external encapsulating structure	GO:0030312	4
cell wall	GO:0005618	45
intracellular	GO:0005622	234
cytoplasm	GO:0005737	386
cytoskeleton	GO:0005856	28
cytosol	GO:0005829	101
endoplasmic reticulum	GO:0005783	152
endosome	GO:0005768	15
Golgi apparatus	GO:0005794	60
lysosome	GO:0005764	36
mitochondrion	GO:0005739	236
peroxisome	GO:0005777	39
plastid	GO:0009536	387
ribosome	GO:0005840	173
vacuole	GO:0005773	47
nucleus	GO:0005634	494
nuclear membrane	GO:0005635	7
nucleolus	GO:0005730	25
nucleoplasm	GO:0005654	6
thylakoid	GO:0009579	78
membrane	GO:0016020	436
plasma membrane	GO:0005886	59
extracellular	GO:0005576	15
extracellular matrix	GO:0005578	6
extracellular space	GO:0005615	8
unlocalized	GO:0005941	11
		
**molecular_function**	GO:0003674	2,391
chaperone activity	GO:0003754	78
catalytic activity	GO:0003824	1,424
hydrolase	GO:0016787	440
kinase	GO:0016301	199
transferase	GO:0016740	427
enzyme regulator activity	GO:0030234	38
binding	GO:0005488	1,278
carbohydrate binding	GO:0030246	1
lipid binding	GO:0008289	22
nucleic acid binding	GO:0003676	540
DNA binding	GO:0003677	245
chromatin binding	GO:0003682	8
transcription factor activity	GO:0003700	63
nuclease activity	GO:0004518	20
RNA binding	GO:0003723	216
translation factor activity, nucleic acid binding	GO:0008135	58
nucleotide binding	GO:0000166	482
oxygen binding	GO:0019825	1
protein binding	GO:0005515	220
molecular_function unknown	GO:0005554	84
motor activity	GO:0003774	17
signal transducer activity	GO:0004871	101
receptor binding	GO:0005102	4
receptor activity	GO:0004872	61
structural molecule activity	GO:0005198	232
transcription regulator activity	GO:0030528	102
translation regulator activity	GO:0045182	58
transporter activity	GO:0005215	332

*P. euphratica *unigene sequences were functionally and structurally annotated using Gene Ontology [22] and the complete assignments were summarized using the plant specific GOSlims [28]. GO terms were derived from matching a unigene sequence to a Swissprot protein using the BLASTX method filtered arbitrarily using the expectation value of 1e-10. Any GO terms associated with the Swissprot entry or keywords were parsed from the Swissprot entry (see Materials and methods for complete details).

**Table 3:** Comparison of DNA microarray data with expression data from quantitative real time PCR

		Array data			Real time PCR repeat 1			Real time PCR repeat 2		
		
GenBank ID	Gene	A	B	C	A	B	C	A	B	C
AJ780552	Cysteine protease	6.3	26.8	17.8	68.4	100.4	365.8	89.9	340.1	130.2
AJ780698	Cyclic nucleotide and calmodulin-regulated ion channel	4.7	8.9	7.7	21	13.6	18.4	15.3	35.3	21.9
AJ780435	Aldehyde dehydrogenase	4.0	4.3	4.8	5.2	4	3.1	6.8	11.2	15.5
AJ769631	ATP-dependent Clp protease ATP-binding subunit clpA	1.6	2.0	2.4	2.1	1.5	1.9	2	1.2	0.8
AJ778655	Alcohol dehydrogenase	0.5	0.7	1.1	0.6	0.6	1.1	0.8	1.1	1
AJ777239	Stable protein/bspA	0.4	0.2	0.5	0.3	0.1	0.03	0.3	0.1	0.05
AJ768555	Dehydration-responsive protein RD22	0.02	0.02	0.02	0.02	0.02	0.03	0.02	0.04	0.03

Two biological repeats were independently reverse transcribed and amplified. The raw threshold cycle (Ct) values were normalized against a glucosidase alpha subunit standard to get normalized ΔCt values, which were used to calculate the fold change in expression between areas A, B, and C compared to area P.

**Table 4:** Metabolite profiling of leaf samples taken from areas A, B, and C and from irrigated controls grown in less saline soil (area P)

	A			B			C			P		
	Average	SE	x fold change from P	Average	SE	x fold change from P	Average	SE	x fold change from P	Average	SE	x fold change from P

Organic acids												
Citric acid	0.442	0.14	*1.59*	0.263	0.07	*0.95*	0.278	0.06	*1.00*	0.278	0.442	0.14
Fumaric acid	0.008	0.00	*1.14*	0.007	0.00	*1.00*	0.007	0.00	*1.00*	0.007	0.008	0.00
Glyceric acid	0.037^a^	0.04	*1.60*	0.023^b^	0.02	*1.00*	0.023^b^	0.02	*1.00*	0.023^ab^	0.037^a^	0.04
Malic acid	0.638	0.15	*0.94*	0.693	0.22	*1.02*	0.669	0.15	*0.99*	0.677	0.638	0.15
Nicotinic acid	0.024	0.00	*0.77*	0.018	0.00	*0.60*	0.027	0.01	*0.87*	0.031	0.024	0.00
Succinic acid	0.381	0.07	*1.10*	0.385	0.08	*1.12*	0.350	0.03	*1.02*	0.344	0.381	0.07
												
Sugars												
Fructose	1.374^b^	0.29	*0.89*	2.326^ab^	0.62	*1.50*	3.394^a^	0.70	*2.20*	1.544^b^	1.374^b^	0.29
Glucose	0.397	0.12	*0.56*	0.697	0.27	*0.99*	0.687	0.47	*0.97*	0.703	0.397	0.12
Raffinose	0.102^b^	0.06	*0.65*	0.309^a^	0.04	*1.98*	0.309^a^	0.08	*1.98*	0.156^ab^	0.102^b^	0.06
Sucrose	610.7	74.70	*0.96*	603.5	63.30	*0.95*	597.2	47.01	*0.94*	633.9	610.7	74.70
Trehalose	0.057	0.01	*0.89*	0.058	0.01	*0.90*	0.062	0.02	*0.97*	0.064	0.057	0.01
Xylulose	0.742^a^	0.05	*1.03*	0.659^b^	0.11	*0.91*	0.662^b^	0.09	*0.92*	0.721^a^	0.742^a^	0.05
												
Sugar-alcohols												
Galactinol	0.087	0.02	*0.63*	0.073	0.03	*0.53*	0.150	0.04	*1.09*	0.138	0.087	0.02
Mannitol	0.368^b^	0.03	*0.99*	0.333^b^	0.04	*0.89*	0.333^b^	0.04	*0.89*	0.371^a^	0.368^b^	0.03
Myo-inositol	2.826^a^	0.10	*1.30*	2.065^bc^	0.27	*0.95*	1.968^c^	0.16	*0.90*	2.170^b^	2.826^a^	0.10
Sorbitol	0.229	0.04	*0.99*	0.210	0.05	*0.70*	0.220	0.03	*0.95*	0.231	0.229	0.04
												
Poly-ol												
Glycerol	0.162^a^	0.05	*6.75*	0.023^b^	0.00	*0.95*	0.024^b^	0.00	*1.00*	0.024^b^	0.162^a^	0.05
												
Amino acids												
β-alanine	0.350^a^	0.03	*2.13*	0.395^a^	0.06	*2.40*	0.121^b^	0.04	*0.74*	0.164^b^	0.350^a^	0.03
Proline	0.045^a^	0.01	*1.67*	0.020^ab^	0.01	*0.75*	0.007^b^	0.00	*0.29*	0.027^ab^	0.045^a^	0.01
Serine	0.014	0.00	*3.50*	0.005	0.00	*1.25*	0.004	0.00	*1.25*	0.004	0.014	0.00
Threonine	0.008	0.00	*1.60*	0.005	0.00	*1.00*	0.005	0.00	*1.00*	0.005	0.008	0.00
Valine	0.041^a^	0.01	*2.40*	0.020^b^	0.00	*1.18*	0.015^b^	0.00	*0.88*	0.017^b^	0.041^a^	0.01

Polar extracts were derivatized and analyzed as described in 'Materials and methods'. Metabolite values were calculated as the relative response ratio (RR) of peak areas of the different compounds with respect to the peak area of ribitol (as internal standard), and are expressed as the average RR/g dry weight of six replicates (normalized with respect to the dry weight (g^-1 ^dry weight). Compounds labeled with a different symbol differ significantly (*p *< 0.05). The fold change relative to trees in area P is indicated in a separate column in italics. This value was calculated from the average values of the metabolites in areas A, B, and C relative to the average value in area P, where P is equal to 1. All leaf samples were harvested on 13 October 2004. Compounds labeled with a different letter (a, b or c) differ significantly (*p *< 0.05). SE, standard error.
