# Supplementary material for: Gene expression and metabolite profiling of Populus euphratica growing in the Negev desert
Source: Genome Biol. 2005 Dec 2;6(12):R101. doi: 10.1186/gb-2005-6-12-r101 (PMC1414072; doi:10.1186/gb-2005-6-12-r101)
Supplement: Additional data file 7 — Microarray analysis of leaf samples taken from areas A, B, C and P in the Ein Avdat valley. [file gb-2005-6-12-r101-S7.DOC]

**Additional data file 7**

| **Microarray analysis of leaf samples taken from areas A, B, C and P in the Ein Avdat valley** | | | | | | | | |
| --- | --- | --- | --- | --- | --- | --- | --- | --- |
| GenBank ID | Annotation | GOSlim biological process | A | *p* value | B | *p* value | C | *p* value |
|  |  |  |  |  |  |  |  |  |
| AJ780423 | Cysteine protease | Protein metabolism | 12.1 | <0.001 | 21.2 | <0.01 | 40.9 | <0.01 |
| AJ780552* | Cysteine protease | Protein metabolism | 6.3 | <0.001 | 26.8 | <0.001 | 17.8 | <0.001 |
| AJ780577 | Cysteine protease | Protein metabolism | 5.0 | <0.001 | 14.3 | <0.001 | 13.8 | <0.001 |
| AJ771356 | Sporulation protein-related | Protein metabolism | 2.7 | <0.001 | 3.2 | <0.001 | 4.3 | <0.001 |
| AJ771208 | Polyubiquitin-like protein | Protein metabolism | 4.5 | <0.001 | 4.6 | <0.001 | 4.1 | <0.01 |
| AJ780294* | Cysteine protease | Protein metabolism | 2.9 | <0.001 | 3.3 | <0.001 | 3.8 | <0.001 |
| AJ779452 | Cysteine protease | Protein metabolism | 2.4 | <0.001 | 2.8 | <0.001 | 3.1 | <0.001 |
| AJ778920 | Putative beta-1,4-N-acetylglucosaminyltransferase | Protein modification | 3.9 | <0.001 | 3.6 | <0.001 | 3.0 | <0.001 |
|  |  |  |  |  |  |  |  |  |
| AJ770288 | Metallothionein type 3b | Response to abiotic or biotic stimulus | 8.8 | <0.001 | 8.6 | <0.001 | 8.8 | <0.001 |
| AJ770693 | Metallothionein type 3b | Response to abiotic or biotic stimulus | 9.9 | <0.001 | 9.7 | <0.001 | 7.3 | <0.001 |
| AJ779297 | Metallothionein-like protein type 2c | Response to abiotic or biotic stimulus | 5.4 | <0.001 | 4.9 | <0.001 | 4.8 | <0.001 |
| AJ773000* | Metallothionein type 3a | Response to abiotic or biotic stimulus | 6.8 | <0.001 | 5.6 | <0.001 | 4.8 | <0.001 |
| AJ772333 | ATP-dependent Clp protease ATP-binding subunit clpA | Response to abiotic or biotic stimulus | 2.8 | <0.001 | 4.6 | <0.001 | 4.5 | <0.001 |
| AJ779386 | Osmotin-like protein | Response to abiotic or biotic stimulus | 3.6 | <0.001 | 2.7 | <0.001 | 3.5 | <0.001 |
| AJ779443 | Metallothionein-like protein type 2c | Response to abiotic or biotic stimulus | 3.0 | <0.01 | 3.7 | <0.001 | 3.3 | <0.01 |
| AJ779694* | Plastid terminal oxidase | Response to abiotic or biotic stimulus | 2.1 | <0.001 | 2.3 | <0.001 | 3.2 | <0.01 |
| AJ771331 | Metallothionein type 3a | Response to abiotic or biotic stimulus | 4.5 | <0.001 | 4.1 | <0.001 | 2.8 | <0.001 |
| AJ774376 | Metallothionein-like protein type 2c | Response to abiotic or biotic stimulus | 4.3 | <0.001 | 4.2 | <0.01 | 2.6 | <0.001 |
| AJ769631* | ATP-dependent Clp protease ATP-binding subunit clpA | Response to abiotic or biotic stimulus | 1.6 | <0.001 | 2.0 | <0.001 | 2.4 | <0.001 |
| AJ778685* | Aluminium induced protein | Response to abiotic or biotic stimulus | 1.6 | <0.001 | 1.7 | <0.001 | 2.3 | <0.001 |
| AJ772175* | Metallothionein type 3b | Response to abiotic or biotic stimulus | 3.2 | <0.001 | 3.2 | <0.001 | 1.9 | <0.001 |
| AJ773042 | Metallothionein type 3a | Response to abiotic or biotic stimulus | 2.8 | <0.001 | 2.2 | <0.01 | 1.7 | <0.01 |
|  |  |  |  |  |  |  |  |  |
| AJ772117 | Putative receptor-like serine/threonine kinase | Signal transduction | 9.4 | <0.001 | 8.7 | <0.001 | 8.0 | <0.001 |
| AJ780698 | Cyclic nucleotide and calmodulin-regulated ion channel | Signal transduction | 4.7 | <0.001 | 8.9 | <0.001 | 7.7 | <0.01 |
| AJ780809 | Cyclic nucleotide and calmodulin-regulated ion channel | Signal transduction | 3.5 | <0.001 | 6.0 | <0.001 | 5.2 | <0.001 |
| AJ769977 | Cyclic nucleotide and calmodulin-regulated ion channel | Signal transduction | 3.1 | <0.001 | 3.6 | <0.001 | 4.3 | 0.297 |
| AJ767463 | Putative phospholipase C | Signal transduction | 3.9 | <0.001 | 4.9 | <0.001 | 3.9 | <0.001 |
| AJ772066 | Cyclic nucleotide and calmodulin-regulated ion channel | Signal transduction | 3.1 | <0.001 | 2.8 | <0.001 | 3.4 | <0.001 |
|  |  |  |  |  |  |  |  |  |
| AJ778911 | Alanine aminotransferase | Amino acid and derivative metabolism | 2.8 | <0.001 | 2.6 | <0.001 | 2.1 | <0.001 |
| AJ769778 | Beta-amylase | Carbohydrate metabolism | 3.3 | <0.001 | 3.5 | <0.001 | 4.8 | <0.001 |
| AJ769912 | Granule-bound starch synthase | Carbohydrate metabolism | 2.9 | <0.001 | 2.0 | 0.105 | 3.0 | <0.001 |
| AJ769227 | Galactinol synthase, isoform GolS | Carbohydrate metabolism | 3.7 | <0.001 | 3.2 | <0.001 | 2.6 | <0.001 |
| AJ767459 | Galactinol synthase, isoform GolS | Carbohydrate metabolism | 3.4 | <0.001 | 3.1 | <0.001 | 2.5 | <0.001 |
| AJ771722 | Transketolase, chloroplast | Carbohydrate metabolism | 2.2 | <0.001 | 2.0 | <0.001 | 2.5 | <0.001 |
| AJ770033 | Ferritin | Ion transport | 3.3 | <0.001 | 4.7 | 0.0158 | 2.9 | <0.001 |
| AJ771629* | Flavonol 3-O-glucosyltransferase | Metabolism | 2.2 | <0.001 | 3.2 | <0.001 | 4.9 | <0.001 |
| AJ780435 | Aldehyde dehydrogenase | Metabolism | 4.0 | <0.001 | 4.3 | <0.001 | 4.8 | <0.001 |
| AJ776096 | Alcohol dehydrogenase | Metabolism | 2.4 | <0.001 | 2.7 | <0.001 | 2.7 | <0.001 |
| AJ768966 | Glutamine synthetase | Metabolism | 1.9 | <0.001 | 2.2 | <0.001 | 2.4 | <0.001 |
| AJ773225 | Photosystem II 44 kDa reaction center protein (P6 protein) | Photosynthesis | 2.0 | <0.001 | 2.2 | <0.001 | 2.5 | <0.001 |
| AJ767241 | Ribulose bisphosphate carboxylase/oxygenase activase | Photosynthesis | 2.1 | <0.001 | 2.2 | <0.001 | 2.4 | <0.001 |
| AJ779100 | S-adenosylmethionine decarboxylase | Secondary metabolism | 2.4 | <0.001 | 2.7 | <0.001 | 2.3 | <0.001 |
|  |  |  |  |  |  |  |  |  |
| AJ771357* | Expressed protein | Biological process unknown | 10.7 | <0.001 | 4.6 | <0.001 | 8.5 | <0.01 |
| AJ771194* | Expressed protein | Biological process unknown | 3.1 | <0.001 | 6.4 | <0.001 | 8.2 | <0.001 |
| AJ770343* | Expressed protein | Biological process unknown | 2.7 | <0.001 | 4.8 | <0.001 | 7.4 | <0.001 |
| AJ780215* | Endomembrane-associated protein | Biological process unknown | 2.8 | <0.001 | 4.7 | <0.001 | 4.0 | <0.001 |
| AJ773744 | 1,4-Benzoquinone reductase-like, Trp repressor binding protein-like | Biological process unknown | 2.7 | <0.001 | 3.9 | 0.136 | 4.0 | 0.0344 |
| AJ780719* | Expressed protein | Biological process unknown | 2.4 | <0.001 | 2.7 | <0.001 | 3.4 | <0.001 |
| AJ780732 | Expressed protein | Biological process unknown | 2.6 | <0.001 | 2.7 | <0.001 | 3.3 | <0.001 |
| AJ778589* | Expressed protein | Biological process unknown | 4.8 | <0.001 | 4.3 | <0.001 | 2.9 | <0.001 |
| AJ780834 | Flower development cycloidea like protein | Biological process unknown | 2.8 | <0.001 | 4.1 | <0.001 | 2.8 | <0.001 |
| AJ776268* | Expressed protein | Biological process unknown | 2.3 | <0.001 | 3.4 | <0.001 | 2.5 | <0.001 |
| AJ778477 | Expressed protein | Biological process unknown | 1.8 | <0.001 | 2.3 | <0.001 | 2.2 | <0.001 |
| AJ769672* | Expressed protein | Biological process unknown | 2.4 | <0.001 | 2.0 | <0.001 | 1.7 | <0.001 |
| AJ769696 | Expressed protein | Biological process unknown | 2.7 | <0.001 | 2.5 | <0.001 | 1.7 | 0.0559 |
| AJ769970* | Expressed protein | Biological process unknown | 2.3 | <0.001 | 2.3 | <0.001 | 1.5 | <0.01 |
|  |  |  |  |  |  |  |  |  |
| AJ778512* | Early light-induced protein | Response to abiotic or biotic stimulus | 0.4 | <0.01 | 0.8 | 0.143 | 1.2 | 0.11 |
| AJ777239* | Stable protein/bspA | Response to abiotic or biotic stimulus | 0.4 | <0.01 | 0.2 | 0.0888 | 0.5 | 0.086 |
| AJ775508 | Peroxidase | Response to abiotic or biotic stimulus | 0.4 | <0.01 | 0.7 | 0.242 | 0.4 | 0.0655 |
| AJ777667* | Chalcone synthase | Response to abiotic or biotic stimulus | 0.1 | <0.01 | 0.1 | <0.01 | 0.3 | 0.0155 |
| AJ773215 | Basic chitinase | Response to abiotic or biotic stimulus | 0.3 | <0.01 | 0.3 | 0.0134 | 0.2 | 0.017 |
| AJ773118 | Dehydration-responsive protein RD22 | Response to abiotic or biotic stimulus | 0.2 | <0.01 | 0.3 | 0.014 | 0.2 | 0.0133 |
| AJ776763 | Chalcone synthase | Response to abiotic or biotic stimulus | 0.1 | <0.01 | 0.1 | 0.0108 | 0.1 | 0.0248 |
| AJ768632 | Lipid transfer protein | Response to abiotic or biotic stimulus | 0.1 | <0.01 | 0.1 | <0.01 | 0.1 | 0.0103 |
| AJ779381* | Wound-induced protein | Response to abiotic or biotic stimulus | 0.3 | <0.01 | 0.1 | 0.0104 | 0.05 | 0.0104 |
| AJ768555 | Dehydration-responsive protein RD22 | Response to abiotic or biotic stimulus | 0.02 | <0.01 | 0.02 | <0.01 | 0.02 | <0.01 |
|  |  |  |  |  |  |  |  |  |
| AJ780862 | Aquaporin (plasma membrane intrinsic protein 2B) | Transport | 0.4 | <0.01 | 0.3 | 0.0146 | 0.3 | 0.0174 |
| AJ772852 | Proline-rich protein/Lipid transfer protein | Transport | 0.2 | <0.01 | 0.3 | 0.0154 | 0.3 | 0.0146 |
| AJ771577 | Putative aquaporin (tonoplast intrinsic protein gamma) | Transport | 0.3 | <0.01 | 0.3 | 0.0137 | 0.2 | 0.0135 |
|  |  |  |  |  |  |  |  |  |
| AJ777296 | Nitrate reductase | Amino acid and derivative metabolism | 0.4 | <0.01 | 0.6 | 0.293 | 0.4 | 0.118 |
| AJ774243 | Trans-cinnamate 4-monooxygenase | Amino acid and derivative metabolism | 0.4 | <0.01 | 0.4 | 0.0303 | 0.4 | 0.0234 |
| AJ774804 | Nucleoid DNA-binding protein cnd41, chloroplast | Protein metabolism | 0.3 | <0.01 | 0.3 | 0.0202 | 0.3 | 0.0156 |
| AJ770884 | Chlorophyll a/b-binding protein | Photosynthesis | 0.4 | <0.01 | 0.4 | 0.0145 | 0.3 | 0.0172 |
| AJ770887 | Chlorophyll a/b-binding protein | Photosynthesis | 0.4 | <0.01 | 0.3 | 0.0135 | 0.3 | 0.0176 |
| AJ775526* | Glutathione S-transferase | Metabolism | 0.3 | <0.01 | 0.4 | 0.0256 | 0.6 | 0.0486 |
| AJ775425 | Proline-rich protein | Cell growth and/or maintenance | 0.2 | <0.01 | 0.3 | <0.01 | 0.2 | <0.01 |
| AJ773383 | Proline-rich protein | Cell growth and/or maintenance | 0.1 | <0.01 | 0.1 | 0.0105 | 0.1 | <0.01 |

Leaves from the indicated areas (A, B or C) were used for transcript profiling. The same control was used for all hybridizations and was prepared from irrigated trees at the Ein Avdat parking lot, where the soil is less saline compared to the valley (area P). The ratio is calculated by dividing the normalized area A, B, or C channel intensity with the area P channel intensity. The ratio is the mean value of 6 measurements (3 biological repeats×2 dyeswap technical repeats). A one-sample Student's *t* test is calculated to test whether the mean normalized expression level for the gene is statistically different from 1.0. The leaf samples were harvested on 27 November 2003. The genes are grouped according to their GO Slim annotation. Genes marked with * displayed statistically significant differences (*p*<0.05) in transcript levels between areas A, B or C using Welch ANOVA as described in “Materials and methods.
